# Assistive tools for classifying neurological disorders using fMRI and deep learning: A guide and example

**DOI:** 10.1002/brb3.3554

**Published:** 2024-06-06

**Authors:** Samuel L. Warren, Danish M. Khan, Ahmed A. Moustafa

**Affiliations:** ^1^ Faculty of Society and Design, School of Psychology Bond University Gold Coast Queensland Australia; ^2^ Department of Electronic Engineering NED University of Engineering & Technology Karachi Sindh Pakistan; ^3^ The Faculty of Health Sciences, Department of Human Anatomy and Physiology University of Johannesburg Auckland Park South Africa

**Keywords:** autism spectrum disorder (ASD), convolutional neural network (CNN), deep learning (DL), disease classification, functional magnetic resonance imaging (fMRI)

## Abstract

**Background:**

Deep‐learning (DL) methods are rapidly changing the way researchers classify neurological disorders. For example, combining functional magnetic resonance imaging (fMRI) and DL has helped researchers identify functional biomarkers of neurological disorders (e.g., brain activation and connectivity) and pilot innovative diagnostic models. However, the knowledge required to perform DL analyses is often domain‐specific and is not widely taught in the brain sciences (e.g., psychology, neuroscience, and cognitive science). Conversely, neurological diagnoses and neuroimaging training (e.g., fMRI) are largely restricted to the brain and medical sciences. In turn, these disciplinary knowledge barriers and distinct specializations can act as hurdles that prevent the combination of fMRI and DL pipelines. The complexity of fMRI and DL methods also hinders their clinical adoption and generalization to real‐world diagnoses. For example, most current models are not designed for clinical settings or use by nonspecialized populations such as students, clinicians, and healthcare workers. Accordingly, there is a growing area of assistive tools (e.g., software and programming packages) that aim to streamline and increase the accessibility of fMRI and DL pipelines for the diagnoses of neurological disorders.

**Objectives and Methods:**

In this study, we present an introductory guide to some popular DL and fMRI assistive tools. We also create an example autism spectrum disorder (ASD) classification model using assistive tools (e.g., Optuna, GIFT, and the ABIDE preprocessed repository), fMRI, and a convolutional neural network.

**Results:**

In turn, we provide researchers with a guide to assistive tools and give an example of a streamlined fMRI and DL pipeline.

**Conclusions:**

We are confident that this study can help more researchers enter the field and create accessible fMRI and deep‐learning diagnostic models for neurological disorders.

## Graphical abstract

In this article, we provide a guide to assistive tools that can simplify and increase the accessibility of fMRI and deep‐learning methods for neurological disease diagnoses. We also detail an example of one such pipeline that can semiautonomously classify autism spectrum disorder.

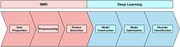

## 1 INTRODUCTION

Deep learning (DL) is an analytical method commonly used to identify, classify, and predict phenomena from data (LeCun et al., 2015). Simply put, DL is a form of modeling that learns patterns from a dataset and then applies that knowledge to a related problem. For example, researchers commonly use it with neuroimaging datasets to distinguish neurodivergent and neurotypical brains (e.g., ASD vs. controls; Ke et al., 2020). Contemporary methods are derived from traditional statistical and machine learning techniques. However, DL differs from traditional techniques due to its basis on human learning (e.g., neural networks; LeCun et al., 2015) and end‐to‐end design (i.e., feature extraction, learning, and classification are all connected within one model). For example, researchers might commonly use DL to analyze a complex problem with a large dataset and traditional methods for linear problems with small datasets (Bzdok et al., 2018; Suzuki, 2017). Accordingly, DL is often paired with neuroimaging datasets due to its ability to analyze complex problems (Noor et al., 2019; Yamanakkanavar et al., 2020). DL is also advantageous for neuroimaging‐base classification because it can analyze high dimensional data, combine neuroimaging sources (e.g., data concatenation and meta‐models), has a high accuracy, and can run unsupervised (Esteva et al., 2019; Raza & Singh, 2021; Wu et al., 2022). Consequently, it is a flexible tool that can be applied to complex research problems, such as classifying neurological disorders.

The combination of DL and neuroimaging methods (e.g., magnetic resonance imaging [MRI]) has rapidly increased in recent years. This combination has resulted in highly accurate models that can reliably classify neurological disorders (Y.‐K. Kim & Na, 2018; Noor et al., 2019). Most of these classification models use structural information from MRI (e.g., brain region size, volume, and thickness). However, other structural modalities, such as positron emission tomography (PET) and computed tomography (CT), are also common (Zhao & Zhao, 2021). These structural models have strong evidence and have the potential for clinical diagnoses. Yet, structural modalities and their biomarkers are not the only methods for measuring neurological disorders. Evidence suggests that functional information, such as brain activation, could be a unique biomarker of disorders like ASD and early‐stage Alzheimer's disease (AD; Feng et al., 2022; Warren & Moustafa, 2023). Harnessing functional biomarkers is important because it can help diagnose disorders not exclusively dependent on structural change (e.g., ASD; Khodatars et al., 2021). Thus, researchers have also investigated the ability of DL to detect neurological disorders when combined with functional measures such as functional MRI (fMRI; Yin et al., 2022). For example, studies have found that fMRI can classify multiple stages of the AD spectrum, such as subjective memory complaints (SMC), mild cognitive impairment (MCI), and late‐stage AD (Parmar et al., 2020). fMRI has also been applied to the classification of other neurological disorders such as ASD, Schizophrenia, depression, and epilepsy (Pominova et al., 2018; Qureshi et al., 2019; Shao et al., 2021); however, this field of fMRI DL research is rather small and requires significant development to be clinically and economically viable.

One overarching reason for the lack of fMRI and DL research is its complexity. fMRI data is not easy to collect, requires significant preprocessing (cleaning), and its analysis requires highly specialized skills (when compared to other neuroimaging methods). While DL does help to streamline the analysis of fMRI data, it also brings its own complexities. Specifically, DL is also a highly complex technique requiring considerable specialized knowledge (e.g., coding, model design, and computer science theory). For example, a typical fMRI and DL classification study may require a researcher to understand big data methods, fMRI acquisition, preprocessing pipelines, coding, model design, hyperparameter optimization, and classification techniques (e.g., Alorf & Khan, 2022). Accordingly, DL methods may seem foreign to neuroimaging researchers as they are rooted in computer science and mathematical concepts not commonly taught in the brain sciences. Equally, DL specialists may find fMRI analyses unfamiliar due to the associated statistical and clinical skills often limited to the brain sciences. However, assistive tools can streamline analyses and, thus, increase the accessibility of fMRI and DL research. In this article, we define assistive tools as methods (e.g., software and programming packages) that can simplify, automate, streamline, or circumvent stages of an fMRI and DL classification pipeline. By using these assistive tools, researchers can optimize their classification pipelines and confidently perform fMRI and DL research. Moreover, by harnessing these assistive tools, researchers could also create pipelines that are more accessible and interpretable for real‐world use (e.g., they can be used by nonspecialized populations such as students, clinicians, and healthcare workers). Such models could help to increase the accessibility and viability of fMRI in both diagnostic research and clinical practice.

Consequently, in this study, we present an introductory guide to assistive tools and techniques that can be used to streamline (e.g., automate and simplify) DL and fMRI pipelines. Specifically, we outline methods for streamlining data preparation, fMRI preprocessing, feature extraction, DL model construction, and model optimization. We exclusively focus on resting‐state fMRI methods because they are the predominant functional method used with DL models; however, many assistive tools are also useful in similar modalities (e.g., task‐based fMRI, magnetoencephalography, and electroencephalography). Following our guide, we detail an example pipeline where we classify ASD using fMRI, DL, and assistive tools. This example aims to give an example of a full pipeline and detail the resources required to execute such a project. To our knowledge, no other study has sought to outline assistive tools for the fMRI and DL‐based classification of neurological disease. It is important to note that this study does not seek to explain the full theory and methods underlying DL and fMRI classification models. These topics have been covered in prior reviews (Feng et al., 2022; Valliani et al., 2019; Warren & Moustafa, 2023; Yin et al., 2022). It should also be stated that this article only introduces assistive tools and is not an exhaustive list of all tools. Instead, we aim to highlight some of the prominent assistive tools for streamlining DL and fMRI classification pipelines and provide an example of one such pipeline.

## 2 GUIDE METHODS

This guide explores common assistive tools that streamline fMRI and DL pipelines for neurological disorder classification. We define assistive tools as any program, package, library, database, or software that increases the speed and decreases the difficulty of creating an fMRI and DL classification pipeline. Relevant sources were obtained using a selection criterion defined by the research team. This selection criterion required that sources are in English (due to language constraints), discuss at least one assistive tool for performing fMRI or DL analysis, and be easily accessible to researchers (e.g., are not private databases). Importantly, the assistive tools did not need to be exclusive to fMRI, DL, or neurological disorder classification. Instead, the assistive tools only needed to generalize to fMRI, DL, or neurological disorder classification research. Unlike a traditional psychological review, we did not exclusively use academic texts because many assistive tools are created and used outside the academic literature. For example, many software packages will have GitHub pages but not academic articles (except for fMRI preprocessing software). Accordingly, we used multiple search engines to find assistive tools. We specifically used Google Scholar, GitHub, papers with code, and arXiv. When a journal article discussed an assistive tool, we used the original source (when possible). Our literature search was restricted to common tools and a predefined time limit of 3 months because of the magnitude of potential tools and sources. All tools were included as long as they met our selection criteria and the scope of this article. Our results are discussed in the following section (Section 3).

## 3 A GUIDE TO ASSISTIVE TOOLS FOR FMRI AND DEEP LEARNING PIPELINES

**FIGURE 1:**
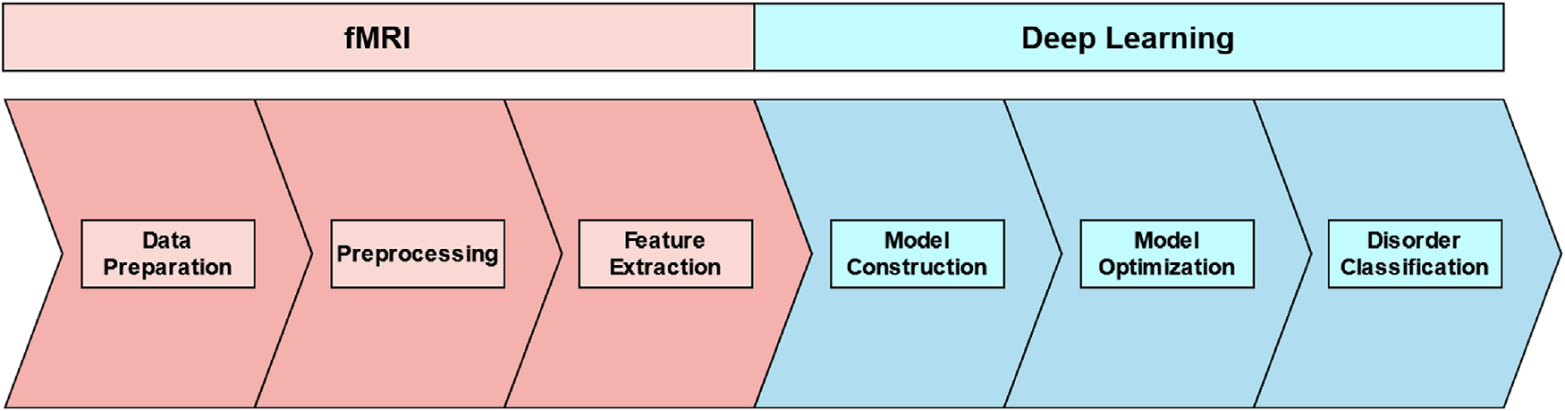
A typical fMRI and deep‐learning pipeline. Note that fMRI and DL both include feature extraction stages. In this paper, we predominately discuss fMRI feature extraction because it is an individual stage of a pipeline. Comparatively, DL feature extraction is almost always contained in the processes of a DL model and, thus, rarely has dedicated assistive tools. It should also be noted that DL model construction and optimization include other substages such as training, validation, and fine‐tuning; however, these stages are not the focus of this article (for similar reasons as DL feature extraction). For more information on DL pipelines, see reviews by Feng et al. (2022), Valliani et al. (2019), and Yin et al. (2022).

A typical DL and fMRI pipeline follows the general stages of fMRI data preparation, preprocessing, feature extraction, model construction, model optimization, and results in disorder classification (see Figure 1). We discuss these stages and their associated tools in the following sections.

### 3.1 Data preparation

**FIGURE 2:**
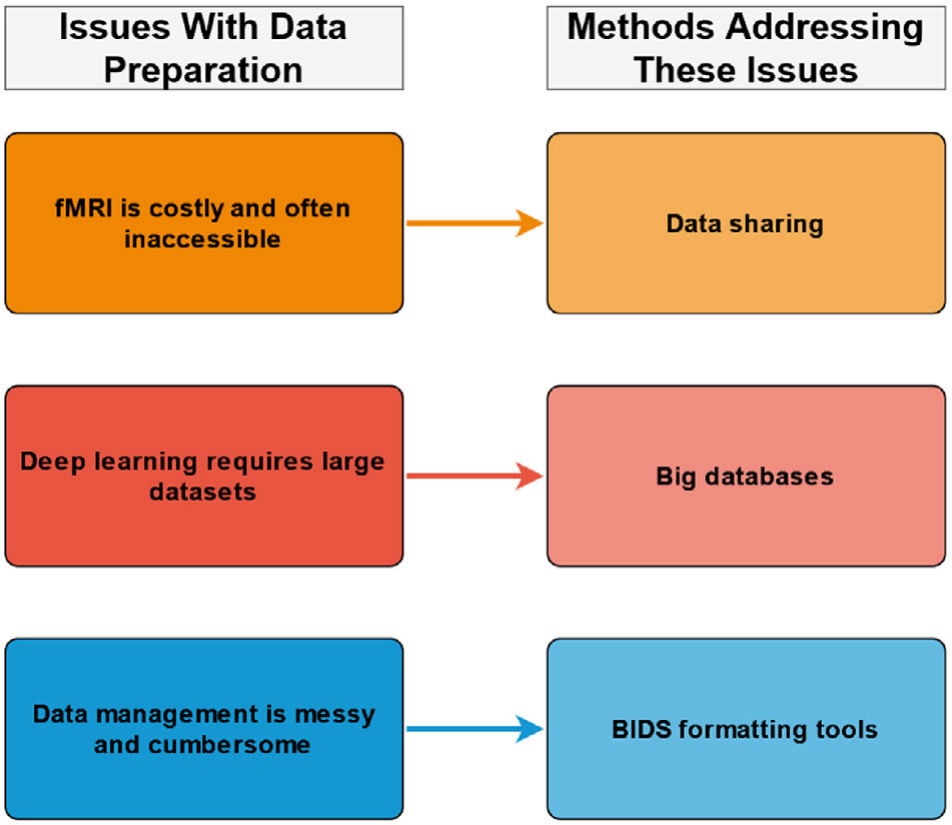
A summary of the problems with data preparation and potential solutions. Note: This figure is only a summary of the general problems and solutions discussed in this article. Many more specific problems and solutions exist concerning data preparation.

Data preparation is the initial stage of an fMRI and DL pipeline (see Figure 1). This stage typically involves the steps of acquiring, cleaning, and organizing fMRI data. Data acquisition is the initial step, where data is manually collected or obtained from a third party (e.g., other researchers, databases, labs, or organizations). Data acquisition may seem trivial; however, a study will change in complexity, scope, and methodology depending on the data acquired. For example, a study classifying binary conditions (e.g., AD vs. control; Chen & Kang, 2023) may require less data and resources than one performing multiclass classification (e.g., AD vs. MCI vs. SMC vs. controls; Lin et al., 2021). Manual fMRI data collection is often seen as the best way to ensure parity between study aims and feasibility. However, fMRI data is inherently complex and takes significant resources to collect (e.g., participants, money, MRI scanner time, and specialist skills). Moreover, the ethics and logistics of fMRI data acquisition are further complicated when studying vulnerable populations (e.g., individuals with dementia). These fMRI data complexities are not made easier with the addition of DL. DL models require significantly more data to classify conditions accurately when compared to traditional fMRI analyses. Accordingly, fMRI data collection can be perceived as too costly for individual researchers or a typical lab, even when not performing DL (see Figure 2 for a summary).

One way to overcome the difficulties with fMRI data collection is to use secondary data (i.e., previously collected data). There are multiple organizations and institutions that maintain large fMRI databases that are accessible to researchers. For example, some big datasets that include fMRI data are the Autism Brain Imaging Data Exchange (ABIDE), the Alzheimer's Disease Neuroimaging Initiative (ADNI), the UKBioBank, SchizConnect, and the Cambridge Centre for Ageing and Neuroscience (Cam‐CAN) (see Table 1 for more information). The accessibility of these datasets changes depending on the organization. For example, hospital and government datasets are often private, while institutional or nongovernmental organization (NGO) datasets commonly require applications. It is also important to note that some organizations will often charge for data access while others are open access (pending an application). Accordingly, every big dataset has different practices, advantages, and disadvantages that should be considered.

**TABLE 1:** A Collection of databases containing fMRI data

Databases	Samples	Citations	Link
Aging Brain: Vasculature, Ischemia, and Behavior (ABVIB)	Control, MCI, & AD	‐	https://adrc.usc.edu/
Alzheimer Biomarkers Consortium‐Down Syndrome (ABC‐DS)	Down syndrome and control	Handen et al. (2020)	https://www.nia.nih.gov/research/abc‐ds
Alzheimer's Disease Neuroimaging Initiative (ADNI)	Control, SMC, EMCI, MCI, LMCI, & AD	Beckett et al. (2015), Petersen et al. (2010), Weiner et al. (2017)	https://adni.loni.usc.edu/
Autism Brain Imaging Data Exchange (ABIDE)	ASD & control	Di Martino et al. (2017, 2014)	https://fcon.1000.projects.nitrc.org/indi/abide/
ABIDE Preprocessed	ASD & control	Cameron et al. (2013)	http://preprocessed‐connectomes‐project.org/abide/
Cambridge Centre for Ageing and Neuroscience (Cam‐CAN)	Adults across the lifespan	Shafto et al. (2014)	https://www.cam‐can.org/
Four Repeat Tauopathy Neuroimaging Initiative	Progressive supranuclear palsy & corticobasal syndrome	‐	https://4rtni‐ftldni.ini.usc.edu/
Frontotemporal Lobar Degeneration Neuroimaging Initiative (FTLD)	Control & frontotemporal dementia	Boeve et al. (2019)	https://www.allftd.org/
The Function Biomedical Informatics Research Network (FBIRN)	Control and schizophrenia	Keator et al. (2016)	https://www.nitrc.org/projects/fbirn/
The International Neuroimaging Data‐Sharing Initiative (INDI) 1000 Functional Connectomes Project	Adults across the lifespan	Biswal et al. (2010), Mennes et al. (2013)	http://fcon.1000.projects.nitrc.org/
OpenNeuro (formerly OpenfMRI)	Multiple datasets are available	Poldrack et al. (2013), Poldrack and Gorgolewski (2017)	https://openneuro.org/
Parkinson's Progression Markers Initiative (PPMI)	Control, Parkinson's disease (PD), & prodromal PD	Parkinson Progression Marker Initiative (2011)	https://www.ppmi‐info.org/
SchizConnect	Control, schizophrenia, & bipolar disorder	Ambite et al. (2015), Wang et al. (2016)	http://schizconnect.org/
UKBioBank	Adults across the lifespan	Sudlow et al. (2015)	https://www.ukbiobank.ac.uk/

EMCI, early mild cognitive impairment; LMCI, late mild cognitive impairment.

There are also general strengths and limitations to big data. For example, big data can enable and focus research on a specific topic. Big data can also enable DL research due to the quantity of data. However, big datasets can also be restrictive as researchers cannot control the specific participants, disorders, measurements, or data quality. It should also be noted that downloading data can be slow and costly depending on one's internet and storage capacities. In some cases, downloading can be simplified using assistive tools such as download managers (see Table 2); however, databases often determine downloading options, and software alone cannot fix some problems (e.g., unstable internet connection). Accordingly, it is important to understand the scope and flexibility of a big dataset before committing to a substantial download. Nevertheless, big datasets are assistive tools enabling researchers to readily access large quantities of fMRI data for DL and neurological disorder classification.

**TABLE 2:** An introductory list of assistive tools.

Category	Tool	Links	Citations
Download managers	WinSCP	https://winscp.net/	‐
	Cyberduck	https://cyberduck.io/	‐
	FileZilla	https://filezilla‐project.org/	‐
BIDS formatting tools	BIDScoin	https://github.com/Donders‐Institute/bidscoin	Zwiers et al. (2022)
	Dcm2niix	https://github.com/rordenlab/dcm2niix	X. Li et al. (2016)
	Dcm2bids	https://unfmontreal.github.io/Dcm2Bids/	‐
	MRIcroGL	https://www.nitrc.org/projects/mricrogl/	‐
BIDS validation tools	Bids‐validator	http://bids‐standard.github.io/bids‐validator/	‐
Preprocessing & feature extraction tools	FSL	https://fsl.fmrib.ox.ac.uk/fsl/fslwiki/	Jenkinson et al. (2012)
	SPM12	https://www.fil.ion.ucl.ac.uk/spm/software/spm12/	Friston et al. (2006)
	FreeSurfer	https://surfer.nmr.mgh.harvard.edu/	Fischl (2012)
	BrainVoyager	https://www.brainvoyager.com/	Goebel et al. (2006, 2006)
	fMRIprep	https://fmriprep.org/	Esteban et al. (2020, 2019)
	GIFT	https://trendscenter.org/software/gift/	Rachakonda et al. (2020)
	CONN Toolbox	https://web.conn‐toolbox.org/	Nieto‐Castanon (2020), Whitfield‐Gabrieli and Nieto‐Castanon (2012)
Deep‐learning libraries	PyTorch	https://pytorch.org/	Paszke et al. (2019)
	TensorFlow	https://www.tensorflow.org/	Abadi et al. (2016)
	Keras	https://keras.io/	
	OpenNN	https://www.opennn.net/	
	neuralnet	https://github.com/bips‐hb/neuralnet	Fritsch et al. (2019)
	Fast AI	https://www.fast.ai/	J. Howard and Gugger (2020)
	PyTorch Lightning	https://lightning.ai/pytorch‐lightning	‐
	Deep Learning Toolbox	https://au.mathworks.com/products/deep‐learning	‐
Code repositories	GitHub	https://github.com/	‐
	Huggingface	https://huggingface.co/	‐
	Papers with code	https://paperswithcode.com/	‐
Pretrained neural networks	ResNet	‐	He et al. (2015)
	VGG	‐	Simonyan and Zisserman (2015)
	MobileNet	‐	A. Howard et al. (2019)
	DenseNet	‐	Huang et al. (2018)
	AlexNet	‐	Krizhevsky et al. (2012)
	VideoResNet	‐	Tran et al. (2018)
	Video S3D	‐	Xie et al. (2018)
Optimization tools	WandB	https://wandb.ai/site	‐
	Optuna	https://optuna.org/	Akiba et al. (2019)
	RayTune	https://www.ray.io/	Liaw et al. (2018)
	Learning rate finder	‐	Smith (2018)
	AutoAugment	‐	Cubuk et al. (2019)

*Note*: Some tools may require dependencies (e.g., docker), and other tools are available. Pretrained models often include multiple iterations and variants.

Data cleaning and organization are the next stages of data preparation. These stages are intertwined as they serve the same purpose of preparing the data for preprocessing. Preprocessing software often requires fMRI data to be presented in a specific way known as Brain Imaging Data Structure (BIDS). BIDS was created to standardize neuroimaging data (Gorgolewski et al., 2016). By organizing all fMRI data in the same way, researchers can ensure that their data can be easily shared, preprocessed, transformed, and analyzed using conventional methods. Raw fMRI data—whether manually collected or acquired from a database—is not always in BIDS. Accordingly, researchers must commonly clean and organize their data into BIDS. The specific details for manually creating a BIDS dataset are beyond this paper's scope, but many good resources are available (see https://bids.neuroimaging.io/). BIDS standardization has enabled the creation of assistive tools for fMRI data formatting. Some popular BIDS formatting tools are BIDScoin (Zwiers et al., 2022) and Dcm2bids (which is based on Dcm2niix; X. Li et al., 2016); however, other assistive tools are also available. We suggest researchers choose a BIDS formatting tool based on their project (e.g., data type, operating system, and software accessibility) and personal preference. For more information on BIDS formatting tools, see Table 2 and each assistive tool's associated documentation. Once fMRI data has been formatted, it should be checked using a BIDS validation tool (see Table 2). After validation, the data is then ready for preprocessing.

### 3.2 fMRI preprocessing

Preprocessing is the next stage of an fMRI pipeline (see Figure 1). This stage involves cleaning fMRI images and separating signal (data of interest) from noise. Unlike some other aspects of fMRI pipelines, preprocessing has long been computerized and semiautomated due to the complex nature of fMRI data. Preprocessing usually uses packages such as FSL, SPM, Freesurfer, BrainVoyager, and fMRIprep (see Table 2). These assistive tools use statistical methods to remove artifacts from fMRI data (e.g., participant movement during the brain scan), standardize brain images, and remove unwanted information (e.g., the skull from an MRI image). These preprocessing methods are semiautomated but often require understanding which methods, settings, and corrections are appropriate for one's data. Some packages may also require supervision, such as a professional screening or validating the preprocessing results (e.g., a neurologist). Alternatively, some packages are automated and only require general quality checking. For example, fMRIprep is automated and requires little‐to‐no specialized oversight (Esteban et al., 2020, 2019). Most preprocessing packages are open‐source and free to use. However, some packages require paid software (e.g., SPM12 requires MATLAB). Preprocessing packages have limitations, such as requiring significant resources and being complicated to set up. For example, fMRIprep requires Linux and can be difficult to set up on Windows computers (when compared to a typical program). In turn, the choice of preprocessing tools depends on a researcher's skills, resources, project, and data.

It is important to note that preprocessing is computationally intensive and can take a significant amount of resources depending on the size of a dataset. This computational intensity does not often restrict individuals’ access to preprocessing methods but can drastically increase the time taken and the quantity of data that can be cleaned. These resource limitations can be overcome using mid‐to‐high‐end personal computers, professional workstations, or cloud computing (e.g., Australian universities can access the ARDC Nectar Research Cloud). Alternatively, some big databases do contain preprocessed fMRI data. However, it should be noted that preprocessed fMRI data is highly rare because it is often niche (i.e., the methods and data are specialized to a specific research project), computationally intensive to create, or cannot be shared due to ethics agreements. One of the most popular preprocessed fMRI databases is the ABIDE preprocessed database (Cameron et al., 2013). The ABIDE I preprocessed database contains approximately 1112 participants spanning the autism spectrum, with fMRI data preprocessed using multiple methods. This database is a good example of an assistive tool that has enabled many studies to streamline ASD classification research (e.g., Shao et al., 2021). Nevertheless, more work must be done to make preprocessed fMRI data accessible and, thus, lower the barriers to fMRI research.

### 3.3 fMRI feature extraction

**FIGURE 3:**
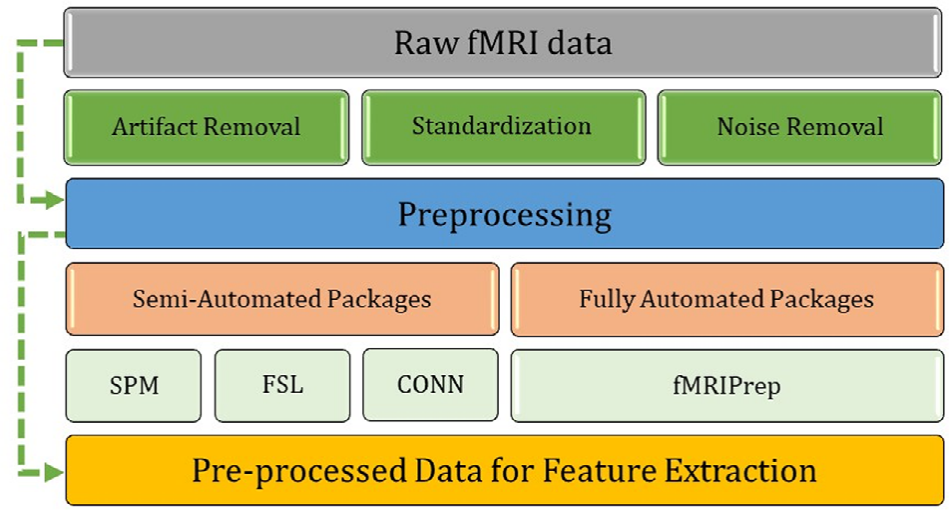
The transformation of raw fMRI data into clean preprocessed data: example steps and tools.

**FIGURE 4:**
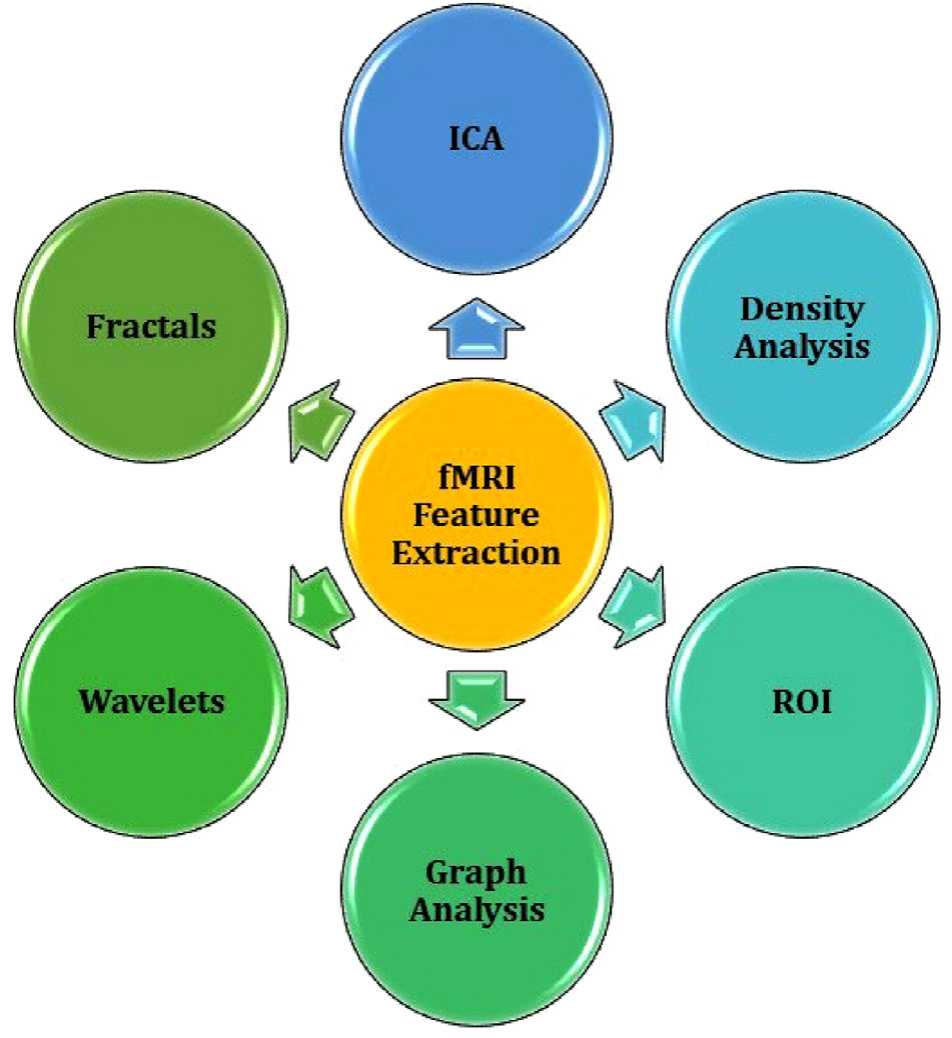
Common fMRI Feature Extraction Techniques. Note: feature extraction techniques are not comprehensively discussed in this article. For more information on these techniques, see Lv et al. (2018), Long et al. (2019), Du and Fan (2013), Du et al. (2020), Campbell et al. (2022), and Efromovich (2019).

fMRI pipelines often require an additional stage of processing known as feature extraction (see Figure 1, 3, and 4). In this stage, key characteristics of the preprocessed data are extracted as variables for analysis. For example, functional connectivity (brain activity) measures are commonly identified from the preprocessed blood oxygen‐dependent (BOLD) signal. These functional connectivity measures can be temporal (e.g., time series), structural (e.g., activation maps), or a combination. Functional connectivity, like preprocessing techniques, is commonly calculated using statistical analyses that distinguish signal from noise. However, unlike preprocessing methods, the resulting features are specific biomarkers chosen based on a study's design and research questions. Some popular feature extraction techniques include region of interest (ROI; e.g., seed‐based correlation) and independent component analysis (ICA; Lv et al., 2018).

ROI methods extract functional connectivity features in a specific brain area, often using a seed‐based approach. ROIs can be determined within a study's population; however, they are more commonly derived from brain atlases (e.g., Automated anatomical labeling atlas [AAL]; Rolls et al., 2020). These seed‐based approaches work by identifying voxels that correlate with the ROI (i.e., seed). These correlations are specifically computed using each voxel's time series (M.‐T. Li et al., 2023). The specific ROIs chosen for analysis ultimately depend on the research question and study design. It is also common for studies to investigate multiple ROIs. For example, Wang et al. (2023) classified AD from controls using ROI methods. Specifically, they identified ROIs using the AAL atlas and then created connectivity matrices using a phase synchronization index approach. The specific ROIs chosen were the cerebellar vermis, cerebellum, temporal lobe, basal ganglia, parietal lobe, limbic system, frontal lobe, and occipital lobe. The matrices for these ROIs were then used in a two‐dimensional (2D) convolutional neural network (CNN) and support vector machine (SVM) model to classify AD from controls with an accuracy of 98.87%. Most studies will pick ROI features based on prior findings in the literature; however, there are also methodological approaches for selecting ROIs. For example, a study by Kim et al. (2023) classified 257 individuals with Attention Deficit Hyperactivity Disorder (ADHD) or controls using an ROI‐based approach. Their fMRI data was acquired from the ADHD‐200 preprocessed repository, and ROI features were extracted using an AAL approach. Kim et al. (2023) extracted 116 ROIs, which they then ranked using an innovative deep‐learning model. They then used a combined CNN and recurrent neural network (RNN) model with the 13 best ROIs to classify ADHD from controls with an accuracy of 70.46%.

An ICA is a data‐driven approach that extracts functional connectivity‐based brain networks using statistical analyses. Rather than using a preexisting atlas, an ICA works by statistically separating distinct fMRI signals in the brain. Depending on the parameters and study design, ICA signals can be included as features or disregarded as noise. For example, a study by Duc et al. (2020) used an ICA to extract multiple brain networks from participants’ fMRI. Their ICA identified 30 independent components, and 16 were kept as relevant features. Some of these components included parts of the visual, cerebellar, attention, auditory‐related, salience, and default mode networks. These features were then input into a three‐dimensional (3D) CNN, which classified AD from controls with a balanced accuracy of approximately 85%. Another study by Ajith et al. (2024) predicted a large cohort of participants’ mental health quality using ICA‐based feature extraction and DL. Specifically, they performed a spatially constrained ICA on 34,606 participants’ data from the UKBioBank. The spatially constrained ICA is an automated approach that works using a reference template. Ajith et al. (2024) used the neruomark template to extract 53 independent components related to the subcortical, sensorimotor, visual, auditory, cognitive‐control, cerebellar, and default mode networks (Du et al., 2020). These ICAs were then transformed into static functional network connectivity matrices and passed into a one‐dimensional (1D) CNN. This model could predict four levels of mental health (poor, fair, good, and excellent) with an average accuracy of approximately 85%.

Feature extraction methods are almost always performed using statistical packages and software. For example, an ICA can be performed using MELODIC, which is a package within FSL (Jenkinson et al., 2012). Like preprocessing methods, functional connectivity calculation requires some oversight (e.g., choosing which parts are noise or signal). However, feature extraction can be semiautomated depending on resource availability, study methodology, and the assistive tools used. For example, an ICA can be automated using a technique known as ICA with reference (e.g., spatially constrained ICA), which identifies fMRI features based on a template (a preexisting map of typical brain networks; Lu & Rajapakse, 2006). It is important to note that different feature extraction methods often identify and calculate features differently. These different feature extraction methods can also produce data in unique formats or measures. Thus, specific feature extraction methods may not fit all research projects and should be considered in the wider context of a study's aims and methodology. For more information on fMRI feature extraction methods, see papers by Lv et al. (2018), Long et al. (2019), Du and Fan (2013), Du et al. (2020), Campbell et al. (2022), and Efromovich (2019). See Table 2 for a list of common assistive tools for feature extraction.

### 3.4 Model construction

The next stage of a DL and fMRI pipeline is model construction (see Figure 1). DL models are constructed using specific programming languages and packages. Most DL packages are built using popular languages like Python, C++, Java, or R. For example, most popular packages—such as PyTorch, TensorFlow, and Keras (see Table 2)—can use Python. DL packages are the primary medium for model creation as they simplify and organize the functions required to perform DL. At the moment, all deep‐learning packages require at least an amateur level of coding knowledge. However, the required knowledge level can drastically change between languages and packages. Learning to use these languages and packages can be quickened by accessing each's forums and learning resources (e.g., tutorials). Often, the more popular a package or language, the easier it is to access tools, help, and resources. Specific research groups also tend to gravitate to certain languages and packages. We suggest that a deep‐learning package or language should be chosen based on a researcher's prior knowledge, study aims, colleagues, and comfort (e.g., a researcher performing preprocessing using Python may choose to create their model in PyTorch).

Some assistive tools can help simplify coding a deep‐learning model. These tools are often known as wrappers because they simplify (wrap) complex code into a simple command. The advantage of these wrappers is that they simplify the coding process and make DL more accessible. The disadvantage of these wrappers is that they are sometimes less flexible than the base DL packages (e.g., tensor flow), and they still require significant knowledge of how to design a model. Some common wrappers include Fast AI, Keras, and PyTorch Lightning (see Table 2). The ability and availability of these wrappers will depend on the language and DL packages used (e.g., the PyTorch Lightning wrapper is limited to the Python language and PyTorch package). These assistive tools are also not a substitution for learning to code or using DL packages. Instead, these wrappers simplify DL packages that can help accelerate model creation without requiring highly specialized knowledge. We recommend that individuals new to the field consider wrappers but also gradually explore the role of all functions within a model. However, learning some coding skills and DL theory are still essential (and are invaluable when creating and interpreting models).

One of the best ways to learn how to construct a DL model is to see how other researchers have coded similar models. It is relatively easy to access code due to the open‐source community. For example, websites like GitHub and papers with code (see Table 2) host many journal articles, scripts, and packages. Combining preexisting code with big datasets (discussed in Section 3.1) can be a good way of learning how to create a model. Another advantage of the open‐source community is the access and distribution of pretrained models. These models are popular architectures created using massive datasets that can be transferred to many problems. Some popular models include ResNet, DenseNet, and AlexNet (He et al., 2015; Huang et al., 2018; Krizhevsky et al., 2012). These pretrained models can reduce the need for building and training a model from scratch. These models can also increase accuracy due to their large training datasets (Han et al., 2021). However, it should be noted that not all pretrained models are instantly compatible with fMRI data, and some model tweaking or data reduction may be required. Nevertheless, pretrained models are great assistive tools for streamlining DL pipelines and classifying neurological conditions (for example, see Meng et al., 2022; Ramzan et al., 2019; Uyulan et al., 2023). See Table 2 for more information on specific pretrained neural networks.

### 3.5 Model optimization and classification

Once a model is written and running, it must be optimized for the best results (see Figure 1). Traditionally, manual optimization involves tweaking the model's settings (i.e., hyperparameters) to increase classification accuracy and reduce loss (error). There are manual methods for searching for the best hyperparameters; however, manual methods are time‐consuming and require significant experience. Alternatively, hyperparameter optimization can be automated using optimization packages (see Table 2). These packages are incorporated into the model's code, changing different hyperparameters and comparing the results to prior runs. These techniques are great for optimizing loss and accuracy, yet they take considerable time and resources. Some popular optimization packages are weights and biases (WandB), Optuna, and RayTune (Akiba et al., 2019; Liaw et al., 2018). Each optimization package has different methods and approaches to hyperparameter tuning (e.g., grid and Bayesian search). Choosing the right method for optimization can take some trial and error. Some manual tuning and domain knowledge are also still required. Nevertheless, these assistive tools can help tune a model's hyperparameters and ensure strong classification accuracy. It is important to note that other emerging methods for automating hyperparameter tuning exist. For example, meta‐optimization is a technique that involves using a second DL model to tune the primary model (Bischl et al., 2023; Jaafra et al., 2019); however, meta‐optimization is relatively new to the field and beyond the scope of this paper. Future research should seek to review emerging assistive tools, such as meta‐optimization.

### 3.6 Guide conclusion

DL and fMRI are not simple methods, yet some assistive tools can help reduce the specialization and time required to create diagnostic models. Such assistive tools cannot replace the need for coding skills and theoretical knowledge. However, assistive tools can help to simplify model creation and disorder classification. It is important to note that this guide is not extensive and that many other assistive tools exist. For example, there are tools for automating data augmentation and models for data generation (e.g., GANS; Qu et al., 2022). Instead, this article only provides an introductory guide to assistive tools for classifying neurological disorders using fMRI and DL. We hope that current and future tools will increase the accessibility of fMRI and DL methods and help scientists make clinically viable diagnostic models for neurological disorders.

## 4 AN EXAMPLE ASSISTIVE TOOLS PIPELINE

Many neurological disorders can be classified using fMRI and DL. For example, ASD is one of the most popular disorders classified in the fMRI and DL literature. ASD is a neurodevelopmental disorder that often manifests as symptoms of social impairment, repetitive behaviors, functional impairment, and intellectual disability (American Psychiatric Association, 2013); however, the presence and manifestation of these symptoms are variable between individuals with ASD and across an individual's lifespan (Wozniak et al., 2017). ASD is commonly paired with functional neuroimaging because disruptions in brain activity and connectivity are key biomarkers of the disorder (Lord et al., 2020). Accordingly, an ever‐expanding literature seeks to diagnose ASD using functional neuroimaging, such as fMRI and electroencephalography (Ayoub et al., 2022). However, these imaging methods are not widely recommended for real‐world diagnoses as they historically perform worse than gold‐standard clinical assessments (Lord et al., 2020). This poor performance of neuroimaging methods occurs for many reasons, including the difficulty of the classification problem (diagnoses are reliant on social constructs and predominantly nonspatial biomarkers) and the variability of an individual's ASD manifestation (as mentioned above). Nevertheless, improvements in ASD diagnosis are required to improve early interventions and, thus, individuals’ quality of life (Lord et al., 2020). In turn, researchers are increasingly turning to complex analytical methods, such as DL, to improve neuroimaging‐based ASD diagnostic methods (S. Li et al., 2022; Zhang et al., 2023). In this article, we create a binary (ASD vs. Control) ASD classification model using fMRI, DL, and assistive tools. This model aims to act as an example of an assistive tools pipeline for new researchers.

### 4.1 Past research

There have been multiple studies that classify ASD from controls using fMRI and DL. These studies predominantly use preexisting data from the ABIDE preprocessed repository. Accordingly, many advancements in ASD diagnoses come from improvements in DL methods. For example, Jönemo et al. (2023) classified ASD from controls using fMRI data and a 3D‐CNN. They acquired their preprocessed data from the ABIDE preprocessed repository, which contained 539 participants with ASD and 573 controls (*N* = 1112). Their study was primarily focused on improving ASD classification accuracy using data augmentation techniques. Data augmentation is a machine learning method that transforms images (e.g., rotation, cropping, and color shifting). Transforming images is beneficial in DL because it artificially increases a dataset's size and can improve a model's classification ability. The data augmentation techniques assessed were image flipping, brightness adjustment, deformation (elastic), rotation, and scaling. Using these techniques, Jönemo et al. (2023) found that they could classify ASD from controls with an accuracy of approximately 62–66%. They also found that data augmentation improved classification by approximately 0.6–2.9%, depending on the technique. However, they could not conclusively recommend a specific augmentation technique because they found that these methods varied in effectiveness depending on design factors (e.g., preprocessing pipeline, features, and dataset). In turn, Jönemo et al. (2023) diagnosed ASD with relatively good accuracy and concluded that data augmentation could improve classification models.

Another avenue for improving ASD classification is the refinement of fMRI feature extraction techniques. For example, Guo et al. (2017) classified ASD from controls using a novel Sparse auto‐encoders‐based (SAE) feature extraction method. Specifically, they took ROI‐based functional connectivity matrices derived from the ABIDE preprocessed repository and extracted features using unsupervised SAE (a type of DL model). These SAE then passed features to a wider deep neural network model that classified ASD from controls. The whole model was trained, validated, and tested using 110 participants from the University of Michigan ABIDE sample. Guo et al's (2017) resulting model could classify ASD from controls with an accuracy of 86%. This classification accuracy is highly accurate compared to other ASD models. However, it should be noted that the literature has observed a difference in classification accuracy between the whole ABIDE dataset and individual data acquisition sites (Heinsfeld et al., 2017). Nevertheless, Guo et al. (2017) showed that strong feature extraction techniques are essential when creating a cutting‐edge ASD classification model.

It is important to highlight that DL model performance varies depending on the ABIDE data used (e.g., the whole dataset or individual data acquisition sites). There is some research into the variance between ABIDE data samples. For example, Zhang et al. (2023) investigated these samples and created a classification pipeline that is reliable across ABIDE acquisition sites. There are seventeen data collection sites in ABIDE I. The associated datasets can vary from one another due to factors like site location, sample size, and individuals’ ASD characteristics. Zhang et al. (2023) achieved parity between ABIDE collection sites using an innovative F‐score feature extraction method that conservatively extracted the best 25% of features from the functional connectivity data (computed using ROI correlations). Their filtered data was then used to train an autoencoder‐based classification model. As a result, their model could classify ASD from controls with an accuracy of 70.9% when using the whole ABIDE dataset. Their model also achieved an average accuracy of 64.5% on each ABIDE data collection site. Accordingly, Zhang et al. (2023) achieved more consistent ABIDE results and, once again, stressed the importance of feature extraction techniques when creating ASD classification models.

### 4.2 The present study

In line with our guide above, this pipeline aims to provide an example of an fMRI and DL pipeline that uses assistive tools. Building on prior studies, we also aim to incorporate contemporary feature extraction methods and data augmentation to maximize our model's classification ability. We aim to show that assistive tools can pair with contemporary methods to create streamlined and competitive models. We also document the tools, data (i.e., which ABIDE samples), time, and resources used to give new researchers an idea of what creating a streamlined pipeline might entail. Accordingly, our aims can be summarized as follows:

1. Create a competitive ASD classification model using fMRI, DL, and assistive tools.
2. Detail the time, resources, and level of automation required for each stage of the pipeline.

The following sections discuss our methods, results, and experiences. These sections follow the pipeline stages outlined in Figure 1 and correspond to each part of the guide above (Section 3).

## 5 MODEL METHODS

### 5.1 Pipeline data preparation

We acquired fMRI data from the ABIDE Preprocessed repository. Our reasoning for using preexisting data can be summarized by the discussion of data acquisition cost and accessibility outlined in Section 3.1 above. Our sample contained 240 ASD and 284 control participants (*N* = 524). The ABIDE preprocessed repository contains more participants; however, the ABIDE II dataset was unavailable at the time of analysis. Our ICA methodology also restricted the usability of some data. Specifically, our ICA required participants’ fMRI data to have the same repetition time (TR). Thus, our sample only included participants with a TR of three (i.e., the largest sample with the same TR data). The exclusion of some participants meant that our sample only contained data from Carnegie Mellon University (CMU), the California Institute of Technology (Caltech), New York University (NYU), San Diego State University (SDSU), Stanford University (Stanford), the University of Michigan (UM), and Yale University (Yale) data collection sites. A further breakdown of our sample's descriptive statistics can be seen in Tables 3 and 4.

**TABLE 3:** Sample categorical descriptive statistics organized by collection site.

Site	Frequency	Percent	Sex (F/M)
Caltech	38	7.25	8/30
CMU	27	5.15	6/21
NYU	184	35.11	37/147
SDSU	36	6.87	7/29
Stanford	40	7.63	8/32
UM	143	27.29	27/116
Yale	56	10.69	16/40

**TABLE 4:** Sample age by collection site.

Site	*n*	Mean	SD	Min	Max
Caltech	38	28.2	10.6	17	56.2
CMU	27	26.6	5.7	19	40
NYU	184	15.3	6.6	6.5	39.1
SDSU	36	14.4	1.8	8.7	17.2
Stanford	40	10.0	1.6	7.5	12.9
UM	143	14.0	3.2	8.2	28.8
Yale	56	12.7	2.9	7	17.8

*Note*: *n =* sample participant count and SD = Standard deviation.

### 5.2 Data preprocessing and feature extraction

Our acquired data was previously preprocessed using the Connectome Computation System (CCS) pipeline via the ABIDE preprocessed repository (as discussed in Section 3.2). This pipeline involves typical preprocessing steps such as slice timing correction, motion realignment, and intensity normalization (Xu et al., 2015). We also used the “filt_global” subcategory of CCS data that had undergone band‐pass filtering and global signal regression. Further details of this preprocessing pipeline can be found on the ABIDE preprocessed website (see Table 1). The ABIDE repository does provide data that is cleaned using other preprocessing pipelines; however, through some preliminary testing, we found that the CCS data was the most compatible with our pipeline (i.e., the data worked well with our ICA). The ABIDE CCS dataset is also commonly used throughout the literature.

**FIGURE 5:**
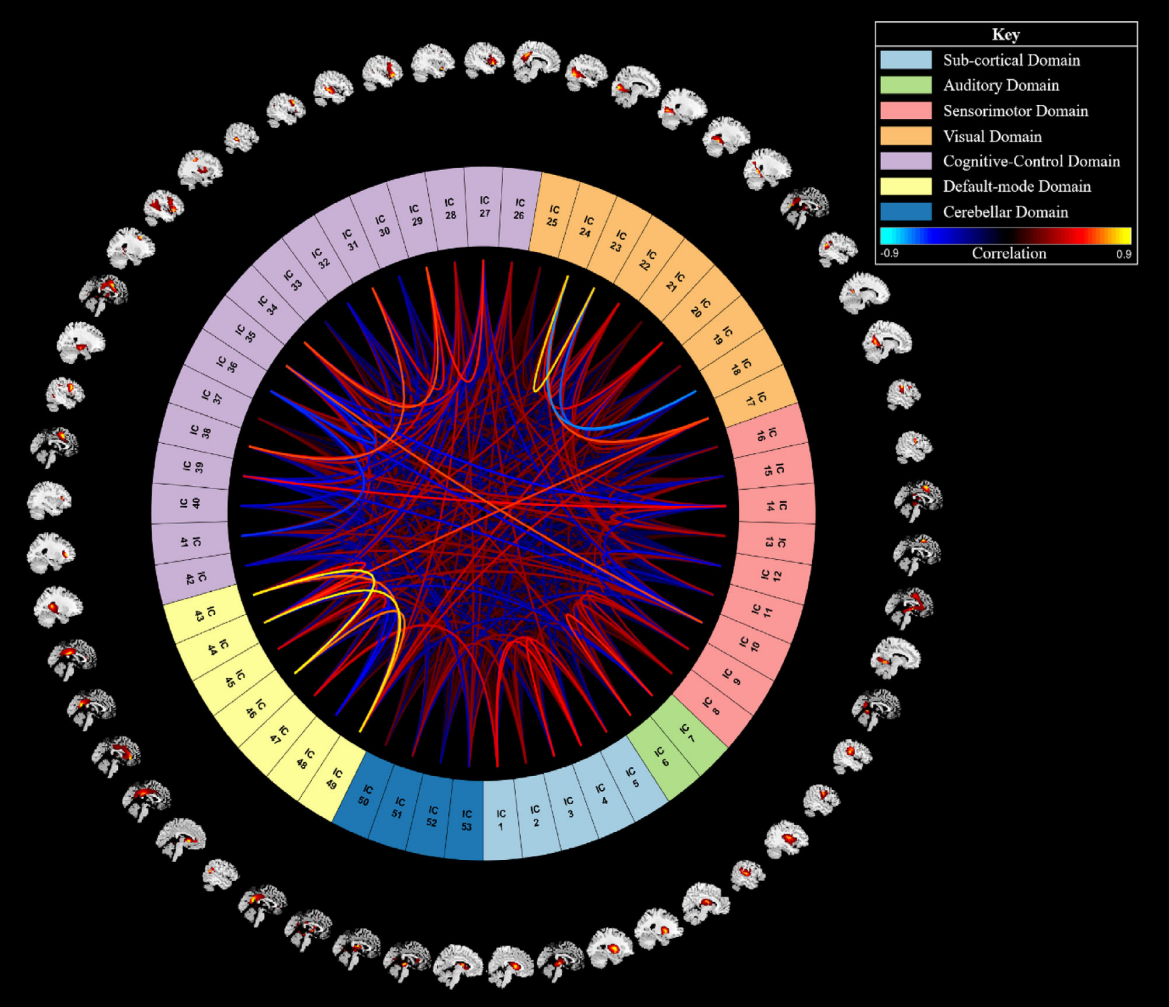
A Connectogram of all 53 networks and 7 domains identified by our GIG‐ICA.

We used a spatially constrained group information‐guided ICA (GIG‐ICA) for feature extraction (see Section 3.3). A spatially constrained GIG‐ICA is a form of group ICA that can automatically identify brain networks using a reference. Our GIG‐ICA was performed using the group ICA of fMRI toolbox (GIFT) in MATLAB R2022a with the default mask setting and the multiobjective optimization with reference algorithm. We also used the NeuroMark template as a reference to identify 53 networks that make up the subcortical, auditory, sensorimotor, visual, cognitive‐control, default mode, and cerebellar domains (Du et al., 2020; see Figure 5). The resulting spatial templates of each participant's networks were then used as features in our deep‐learning classification model.

### 5.3 Model creation and classification methods

**FIGURE 6:**
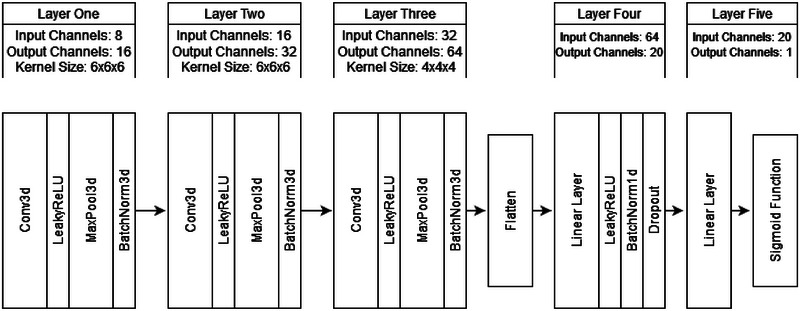
3D‐CNN model architecture. Note: Convolutional layers had a stride of one, and maxpooling layers had a stride of two. No padding was used. The key terms are as follows: inputs = the number of features fed into a layer; outputs = the number of features fed out of a layer; kernel size = the size of a patch that a convolutional layer views and reduces at a time; stride = the amount of units a convolutional kernel moves when viewing parts of an image; padding = adding blank values around an image; maxpooling = a feature extraction and dimensional reduction technique; LeakyReLU = an activation function which allows for minor negative values; batchnorm = normalizes feature values for each batch; dropout = a regularization function that turns a percentage of feature values to 0; Conv3d = a 3D convolutional layer that learn features; linear layers = layers that learn and fit features using a linear function; flatten = a function that dimensionally reduces the data; and sigmoid = a function that turns a feature value into a binary value.

**FIGURE 7:**
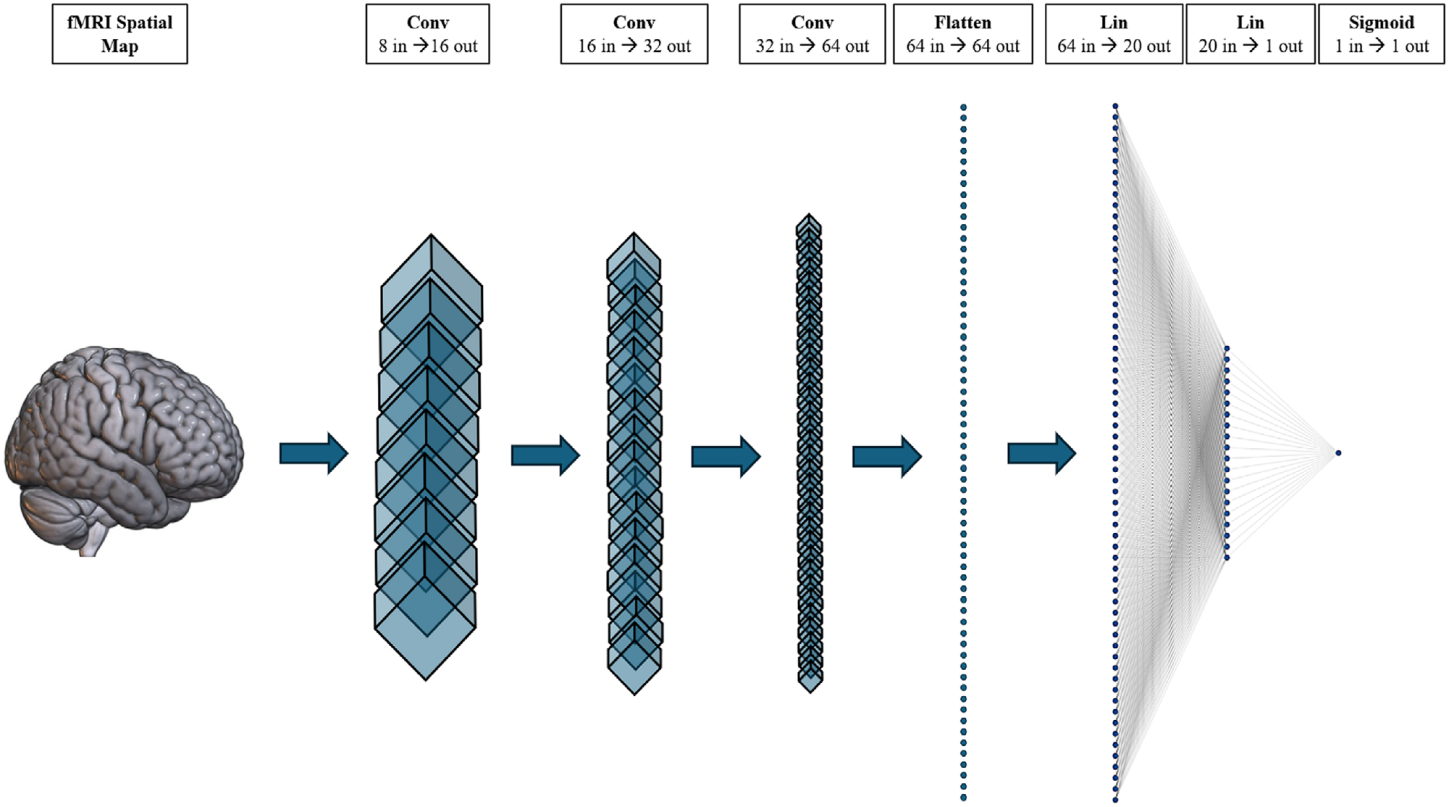
A graphical visualization of our models architecture. Our model contains four key sections: the convolutional layers (Conv), flatten function, linear layers (Lin), and sigmoid classification function. The first convolutional layer takes the fMRI as input, learns the 3D features, and passes them to the next convolutional layer. The remaining convolutional layers continue this trend, gradually learning and extracting key features from the fMRI. Once completed, the flatten function takes the 3D fMRI features and dimensionally reduces the data to one dimension (1D). This 1D data is the passed to the linear layers that continue to learn and fit the features. Finally, the last linear layer reduced the features to one value that is then passed to a sigmoid function for binary classification (i.e., a final value > 0.5 = class 1 and < 0.5 = class 0).

We used a 3D‐CNN to classify ASD using participants’ GIG‐ICA Spatial maps as input. We chose a 3D‐CNN due to the modality and dimensionality of our data. 3D‐CNNs are also widely used in the literature for ASD and similar fMRI classification problems (for example, see Thomas et al., 2020). We constructed our 3D‐CNN using Python and PyTorch (see Section 3.4). These methods were chosen due to our familiarity with the language. Our architecture was loosely based on a version of C3D. C3D is a popular 3D CNN that pairs 3D convolutional layers with batch‐normalization and maxpooling layers to help learn spatial features (Tran et al., 2015). Unlike C3D, our simplified architecture only contained three convolutional and two linear layers. This simplification occurred due to our small dataset and the difference in classification task (i.e., binary classification is often less difficult than multiclass classification). We also substituted the SoftMax classification layer with a sigmoid activation function due to the nature of our binary classification task. Further details of our architecture can be seen in Figures 6 and 7. Our model was initially optimized using Optuna and then underwent manual tuning (see Section 3.5 for a guide to optimization). Further details on our optimization workflow are discussed in the results section below (Section 4.2).

We partitioned our data using a stratified sampler into 80% training, 10% validation, and 10% testing datasets. Our model was trained using the Adam optimizer, binary cross‐entropy loss, a batch size of 52, and a learning rate of 0.0001. Our data also underwent normalization and resizing transformations to improve accuracy and compatibility. Initially, our model experienced significant overfitting due to our small dataset (*N* = 524). We used dropout (*p* = .5) as regularization, 90° random rotation (*p* = .5) for data augmentation, an exponential learning rate scheduler (gamma = 0.9), and an early stopping function (patience = 15) to help manage the overfitting. We opted to use the Medical Open Network for AI (MONAI) rotation transform as this augmentation is specially made for medical images and is compatible with 3D data (The MONAI Consortium, 2020). Our regularization and augmentation methods helped combat overfitting and maximize model accuracy. The final model was trained on a PC using an AMD Ryzen 5 2600 CPU, RTX 3060 (12Gb) GPU, 32Gb of RAM, and Windows 10.

### 5.4 Additional outcomes

Besides model metrics, we also tracked the tasks, tools, and hours taken to complete our DL model. We chose to include these additional metrics to provide new researchers with more details about the timeline and construction of our example pipeline. These statistics were recorded manually using written notes. Our procedures and experiences with each tool were also documented throughout. These notes and statistics were not overly quantitative but aimed to give an approximate summary of creating an assistive tools‐based classification pipeline.

## 6 MODEL RESULTS

### 6.1 ASD classification

**FIGURE 8:**
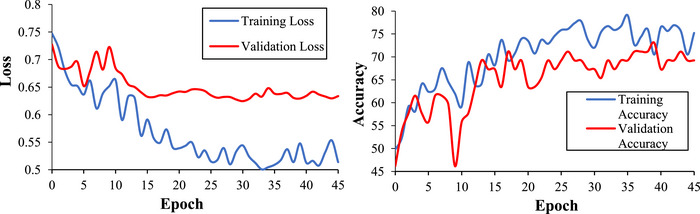
Model training and validation loss and accuracy.

**FIGURE 9:**
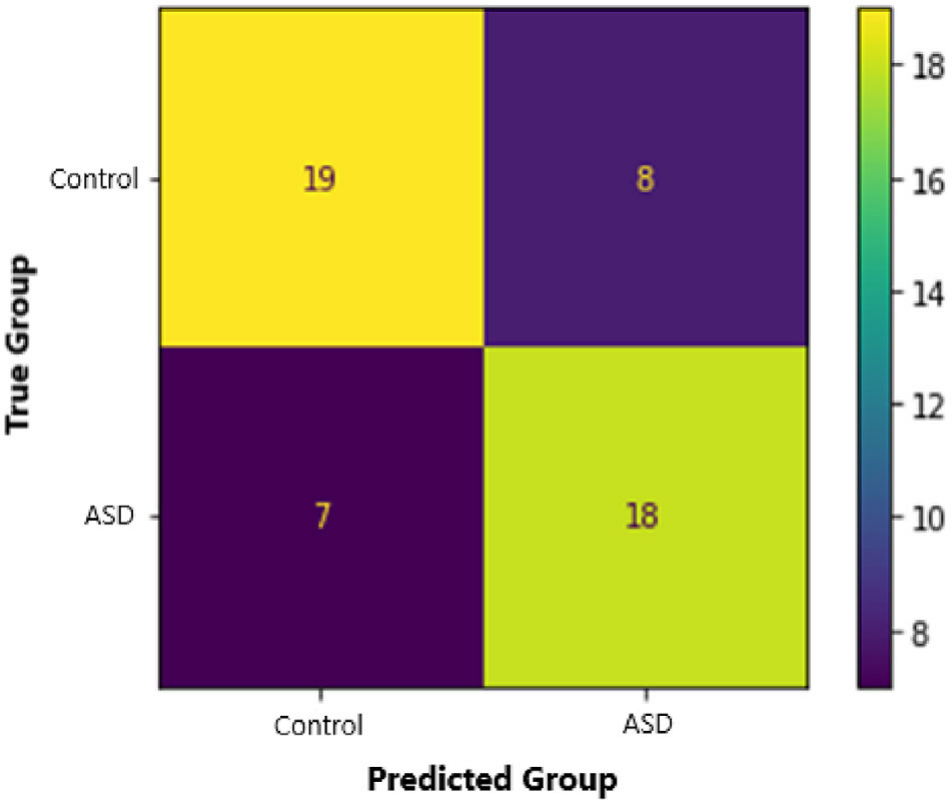
A confusion matrix of the testing dataset's classification results. Note: A confusion matrix displays the relationship between true diagnoses and a model's predicted diagnosis. The values in the top left quadrant are true negatives (controls classified as controls by the model), the top right are false positives (controls classified as ASD), the bottom left are false negatives (ASD classified as controls), and the bottom right are true positives (ASD classified as ASD). The heatmap indicates the frequency of results in each quadrant. A highly accurate model will maximize true values and minimize false values.

Our 3D‐CNN classified ASD participants from controls with an accuracy of 71.2%. This model also had a sensitivity of 72%, a specificity of 70.4%, a precision of 69.2%, and an F1 score of 0.71. We calculated the Matthews Correlation Coefficient (MCC) to understand the relationship between predicted and true diagnoses. MCC is a binary classification correlation coefficient that is based on and interpreted similarly to Pearson's correlation (Chicco & Jurman, 2020; Matthews, 1975). Our MCC was 0.42, indicating a strong positive relationship between true diagnoses and model predictions. See Figures 8 and 9 for more training, validation, and testing metrics. Our model also performed well compared to contemporary models (see Table 5). For example, our model had the second‐highest accuracy compared to a sample of models using multisite ABIDE datasets. Our model also performed well when compared to other 3D‐CNN models. Consequently, we achieved our aim of creating a competitive ASD classification model using fMRI, DL, and assistive tools.

**TABLE 5:** Comparing our model to similar ABIDE samples and model architectures.

Paper	ABIDE sample	Sample size (*N*)	Data split	Model type	Accuracy
Deng et al. (2022)	Full ABIDE I Dataset	1112	5‐fold CV	3D‐CNN	75%
X. Yang et al. (2020)	Full ABIDE I Dataset^a^	1035	5‐fold CV	Multilayer perceptron	75%
**Our model**	Half of ABIDE I	524	80/10/10^b^	3D‐CNN	71%
Zhang et al. (2023)	Full ABIDE I Dataset	1112	10‐fold CV	Autoencoder	71%
Heinsfeld et al. (2017)	Full ABIDE I Dataset^a^	1035	10‐fold CV	Autoencoder	70%
Jönemo et al. (2023)	Full ABIDE I Dataset	1112	70/15/15^b^	3D‐CNN	66%
Thomas et al. (2020)	Portions of ABIDE I & II	1162	5‐fold CV	3D‐CNN	64%
T. Yang et al. (2022)	Full ABIDE I Dataset^a^	1035	10‐fold CV	GCN	64%
Guo et al. (2017)	The University of Michigan	110	5‐fold nested CV	Autoencoder	86%
T. Yang et al. (2022)	The Olin Neuropsychiatry Research Center	34	LOSO	GCN	76%
Bengs et al. (2020)	New York University	184	70/15/15^b^	3D‐CNN‐RNN	67%

CV, cross‐validation; LOSO, leave one site out.

^a^ Participants with missing phenotypic data were removed, or the dataset was balanced.

^b^ The data split is in the order of training, validation, and testing percentage.

### 6.2 Project workflow and resources

We documented our procedures throughout to provide more details on the assistive tools used and our workflow. The whole project took approximately 6 months and included model construction, learning, troubleshooting, training, metrics, and optimization. Our specific workflow is as follows: First, we downloaded the full ABIDE preprocessed dataset from the preprocessed connectomes project using the Cyberduck download manager. We also downloaded participants’ demographics for classification labels in our 3D‐CNN. Next, we used 7‐Zip to extract all NII files from their compressed state to be compatible with GIFT. We also cleaned the demographic files in Excel and checked for missing data. Then, we downloaded the NeuroMark template (from https://trendscenter.org/data/) and ran the GIG‐ICA using GIFT. We then checked the ordering of the spatial maps and recoded our demographics to pair with GIFT's participant naming scheme. After completing preprocessing and feature extraction, we moved on to DL. We started by finding code online that was similar to our intended design. This search used GitHub, papers with code, and Google Scholar (i.e., reading journal articles). After finding a model architecture and some boilerplate code, we rewrote everything to fit our specific classification problem. This rewriting involved reworking the data loader to be compatible with our data, including stratification, and coding in numerous functions (e.g., Optuna, early stopping, model metrics, and validation). Once the code was functional, we used Optuna to search for hyperparameters that achieved reasonable accuracy (e.g., ∼65%). We also experimented with other packages, such as LRFinder and PyTorch lightning; however, they did not end up in the final model because other tools performed the same function (e.g., Optuna was used to find the best LR instead of LRFinder). We then moved on to manually tuning the hyperparameters to maximize model accuracy. After the model was tuned, we ran and validated our model and extracted the classification metrics. Altogether, these tasks took approximately ten weeks and almost 3 months of full‐time work. A breakdown of these tasks and their approximate time can be seen in Table 6.

**TABLE 6:** A breakdown of the project's main tasks, their hours to completion, and level of automation.

Tasks	Tools	Automation level	Time (hours)
Initial research & project design^a^	Research Databases (e.g., PubMed)	Manual	80^b^
Data download	Cyberduck	Automated	7
File extraction	7‐Zip	Automated	3
Data cleaning & organization	Excel & Windows 10	Manual	3
GIG‐ICA	GIFT	Automated	2
Model & coding research	GitHub & papers with code	Manual	20
Code writing/rewriting	Spyder IDE, Python, & PyTorch	Manual	40
Model reworking & design	Spyder IDE, Python, & PyTorch	Manual	40
Hyperparameter search	Optuna	Automated	120
Manual tuning	Spyder IDE, Python, & PyTorch	Manual	80
Collating metrics and results	Sklearn, Matplotlib, and Word	Manual	4
Total			399

^a^ Project design included selecting assistive tools, finding data, structuring our pipeline, deciding on the classification problem, and outlining the aims for this project.

^b^ *Note* that while the project started with 80 h of research and design, these stages are forever ongoing but hard to quantify. Automated tasks also required a small level of setup and occasional supervision.

It is important to note that the time estimates in Table 6 do not always account for some vague but essential parts of model creation, such as learning to use programs, general data work, troubleshooting, piloting, exploration, and installing dependencies. We do not explicitly report timeframes for these tasks as they are often subjective and hard to define (e.g., does learning to use a program ever end?). Similarly, it is important to note that our timeframes are researcher and technology specific. For example, one's computer speed and capacity (e.g., Memory, CPU thread count, CUDA cores, and SSD speed) will affect the time it takes to run a model. Equally, a researcher's skills and familiarity with a pipeline will also influence the time to perform most tasks. It should also be noted that some tasks can be performed in unison thanks to automation. While somewhat contextual, we hope this example pipeline can help new researchers understand the workflow and potential timeframes for creating an fMRI and DL model. We also hope that it shows the potential of assistive tools to automate and streamline such a pipeline.

## 7 DISCUSSION

In this project, we built an example ASD classification model using fMRI, DL, and assistive tools. Unlike prior studies, we explicitly aimed to incorporate assistive tools to decrease the difficulty of constructing our model and increase the accessibility of our pipeline. In turn, we found that our assistive tools primarily helped us to automate laborious tasks and streamline the stages of our pipeline. For example, we could automate hyperparameter searching (one of the longest stages) and skip steps like data collection and preprocessing. Our resulting model could classify ASD from controls with an accuracy of 71%. This accuracy is highly competitive compared to similar models using multisite data from ABIDE. For example, Deng et al. (2022) achieved an accuracy of 75%, while Jönemo et al. (2023) and Thomas et al. (2020) achieved an accuracy of approximately 65% on similar samples (i.e., multisite ABIDE participants). Accordingly, we showed that cutting‐edge models can be made for ASD classification using fMRI, DL, and assistive tools.

Our model is relatively unique compared to other fMRI and DL ABIDE classification models in the literature. For example, we used a spatially constrained GIG‐ICA with reference instead of common ROI methods. We also used automated techniques like Optuna's hyperparameter optimization search. We selected these alternative methods because of our focus on using assistive tools. For example, our GIG‐ICA helped us streamline our fMRI pipeline by automating the feature extraction stage. This focus on assistive tools may seem contrary to the typical approach of prioritizing model performance (e.g., maximizing classification accuracy); however, our preliminary results suggest that assistive tools do not drastically compromise model performance and could make fMRI and DL classification techniques more accessible to new researchers and clinicians.

It is important to note that our study had some notable strengths and limitations. First, we believe that this guide and example‐model format can be a beneficial learning tool for new researchers. Specifically, our guide can act as a compendium of common assistive tools, while our example model can act as a template for creating a streamlined classification pipeline. Second, it is essential to highlight that our example pipeline is highly contextual, and the specific methods used may not generalize to all classification problems in the field. We want to stress that most projects’ specific timeframes and assistive tools will vary. For example, our project required more hyperparameter tuning time than usual because of our overfitting problems (which are common in small datasets). Nevertheless, our pipeline can still act as an example that can temper expectations and inspire the adoption of assistive tools. It is also important to note that there is an inherent data loss when compacting an entire project into some metrics and a written summary. Not all work leads to results, and model work can sometimes be more of a craft than a science. We hope our example pipeline can help new researchers understand the resources and skills required to create a simple DL and fMRI classification model.

Regarding our model, it is important to highlight that our focus on assistive tools did result in some difficulties that should be considered (e.g., overfitting and sample size restrictions). For example, using a more traditional technique like ROI feature extraction could have resulted in more data and less overfitting. By preferencing assistive tools, we also chose methods and a model architecture that were simplistic when compared to some cutting‐edge techniques. These differences might make it harder to compare our model to other pipelines that use different sample sizes and techniques (e.g., autoencoder models). Our choice to use the ABIDE dataset may have also limited our study as its general accuracy is known to be lower than other neurological datasets. Initially, we wanted to conduct our project on Alzheimer's disease classification and the ADNI dataset; however, this smaller and un‐preprocessed dataset was not feasible for the timeframe of this project. These problems with small datasets also require more complex techniques and attention that are beyond the scope of this paper.

## 8 CONCLUSION

This study is only an introductory guide to assistive tools and an initial proof of concept for an assistive tools pipeline. In turn, future research should seek to expand on this work by applying assistive tools to various aspects of fMRI and DL pipelines. For example, future research could apply assistive tools to cutting‐edge models, incorporate contemporary techniques to assistive tools pipelines (e.g., transfer and ensemble learning), embrace graphical user interface (GUI) DL methods, and increase the accessibility of preexisting preprocessed data. We believe such research can help welcome new researchers into our interdisciplinary field and increase the viability of neurological diagnostic models. More generally, we also believe that improved accessibility can help to increase our ability (as a community) to make clinically viable classification models for neurological disorders. These models could be crucial to improving early diagnoses, treatment, and individuals’ quality of life. DL and fMRI have a long way to go before diagnostic models can be clinically viable. However, we believe that the tools and knowledge required to create clinically viable models are already being created.

## AUTHOR CONTRIBUTIONS

**Samuel L. Warren**: Conceptualization; investigation; writing—original draft; methodology; visualization; writing—review and editing; formal analysis; project administration; data curation; validation; software; resources. **Danish M. Khan**: Visualization; writing—review and editing. **Ahmed A. Moustafa**: Writing—review and editing; conceptualization; supervision.

## FUNDING

This study would like to acknowledge funding received as part of the Australian Government's Research Training Program Scholarship.

## CONFLICT OF INTEREST STATEMENT

The authors of this study declare no conflicts of interest.

### PEER REVIEW

The peer review history for this article is available at https://publons.com/publon/10.1002/brb3.3554

## Data Availability

The data for this study was acquired from the ABIDE preprocessed repository. This data is freely and openly available via their website at http://preprocessed‐connectomes‐project.org/abide/.
